# Quantitative morphometric analysis of adult teleost fish by X-ray computed tomography

**DOI:** 10.1038/s41598-018-34848-z

**Published:** 2018-11-08

**Authors:** Venera Weinhardt, Roman Shkarin, Tobias Wernet, Joachim Wittbrodt, Tilo Baumbach, Felix Loosli

**Affiliations:** 1 Institute for Photon Science and Synchrotron Radiation, Karlsruhe Institute of Technology, Hermann-von-Helmholtz-Platz 1, Eggenstein-Leopoldshafen, Germany; 2 Centre for Organismal Studies, COS, Heidelberg University, Im Neunheimer Feld 230, Heidelberg, Germany; 3 Laboratory for Applications of Synchrotron Radiation, Karlsruhe Institute of Technology, Karlsruhe, Germany; 4 Institute for Applied Computer Science, Karlsruhe Institute of Technology, Karlsruhe, Germany; 5 Institute of Toxicology and Genetics, Karlsruhe Institute of Technology, Karlsruhe, Germany; 6 Present Address: Center for Biological Systems Analysis, Freiburg, Germany

## Abstract

Vertebrate models provide indispensable paradigms to study development and disease. Their analysis requires a quantitative morphometric study of the body, organs and tissues. This is often impeded by pigmentation and sample size. X-ray micro-computed tomography (micro-CT) allows high-resolution volumetric tissue analysis, largely independent of sample size and transparency to visual light. Importantly, micro-CT data are inherently quantitative. We report a complete pipeline of high-throughput 3D data acquisition and image analysis, including tissue preparation and contrast enhancement for micro-CT imaging down to cellular resolution, automated data processing and organ or tissue segmentation that is applicable to comparative 3D morphometrics of small vertebrates. Applied to medaka fish, we first create an annotated anatomical atlas of the entire body, including inner organs as a quantitative morphological description of an adult individual. This atlas serves as a reference model for comparative studies. Using isogenic medaka strains we show that comparative 3D morphometrics of individuals permits identification of quantitative strain-specific traits. Thus, our pipeline enables high resolution morphological analysis as a basis for genotype-phenotype association studies of complex genetic traits in vertebrates.

## Introduction

Genotype-phenotype association is of fundamental importance for many studies, for example etiology of disease. Such associations are facilitated by small tissue volume and transparency to visual light. These criteria are exquisitely met by small teleost models such as medaka (*Oryzias latipes*) and zebrafish (*Danio rerio*)^1,2^. Small and completely transparent embryos allow high resolution analysis using confocal and wide-field light microscopy. More challenging are detailed studies of adult morphometrics, where tissue size and pigmentation often preclude analysis based on visible light. Furthermore, quantitative trait analysis requires precise measurements of morphometrics of the entire body at high resolution. In view of the rapidly increasing use of these genetic models to study disease^2–6^ it is of importance to provide imaging approaches that permit quantitative morphometrics of entire adults.

Previous quantitative phenotype studies did not use whole-body morphometrics, but rather relied on landmark-based approaches with linear measurements to partition traits into different components^7^. Such approaches are limited by the resulting lack of a description of the entire anatomical phenotype in three-dimensions (3D). Recently, the potential of 3D imaging to reveal quantitative morphological phenotypes was demonstrated using optical projection tomography, X-ray micro-computed tomography (micro-CT) and high-resolution episcopic microscopy in mice^8^. Presently X-ray tomography seems to be the best method among available 3D imaging techniques with respect to penetration power, attainable spatial resolution and scanning time. X-ray tomography is a versatile tool for imaging at various resolution scales, ranging from cm to µm voxel sizes and down to nm in X-ray microscopy. Similar to visible light imaging, multiple contrasts, such as absorption, phase and dark-field contrasts are available for visualisation^9,10^. The low natural X-ray absorption of biological soft tissues can be circumvented by X-ray phase contrast at the cost of lower resolution^11,12^. For many applications however, chemical contrast agents can be applied to enhance absorption of X-rays, which unlike phase contrast does not require additional X-ray optical components. Absorption-imaging with the help of contrast agents can be optimised to visualize a wide range of biological tissues and organs, thus enabling a broad spectrum of applications^13,14^.

A few morphometric studies have been reported using X-ray micro-CT in fish, mainly in rainbow trout and zebrafish^15–17^. Due to low differential contrast of soft tissues and spatial resolution, morphometric analysis relied on manual or unsegmented data. Recently, advances in image processing for ultrasonic, magnetic resonance, X-ray and other imaging techniques have been achieved^18,19^. Manual segmentation is tedious and time-consuming, thus semiautomatic and automatic segmentation methods have been developed. Typically they are specifically designed for an organ of interest^20,21^. Whole body anatomy segmentation is too complex for most segmentation. Recently, automatic segmentation of whole body anatomy was successfully performed in mice^22,23^.

Here, we report on the development of an automated micro-CT based image recording and analysis pipeline, in our particular case focusing on small teleosts such as medaka and zebrafish. To ensure applicability of the methodology to comparative morphometrics with large numbers of individuals, we optimized our pipeline also with respect to the chosen spatial resolution and tissue contrast in such a way that it enables application of suitable algorithms for automated segmentation of 3D data and subsequent quantitative morphometric analysis of adult teleosts.

We demonstrate the general application potential of such a pipeline for morphometric analysis of small vertebrates by exemplarily establishing an interactive 3D atlas of an adult medaka fish that permits virtual visualisation of body and organs, followed by comparative quantitative morphometric characterization of different isogenic inbred lines of medaka. This comparative analysis is then used to detect strain specific quantitative traits. We show the particular methodology to be equally well applicable to zebrafish. Hence, the overall procedure is providing a general tool for high resolution anatomical studies for small vertebrates. Our approach thus paves the way for quantitative genotype-phenotype associations of small vertebrate models and by that enables studies of complex genetic traits.

## Results

**Figure 1:**
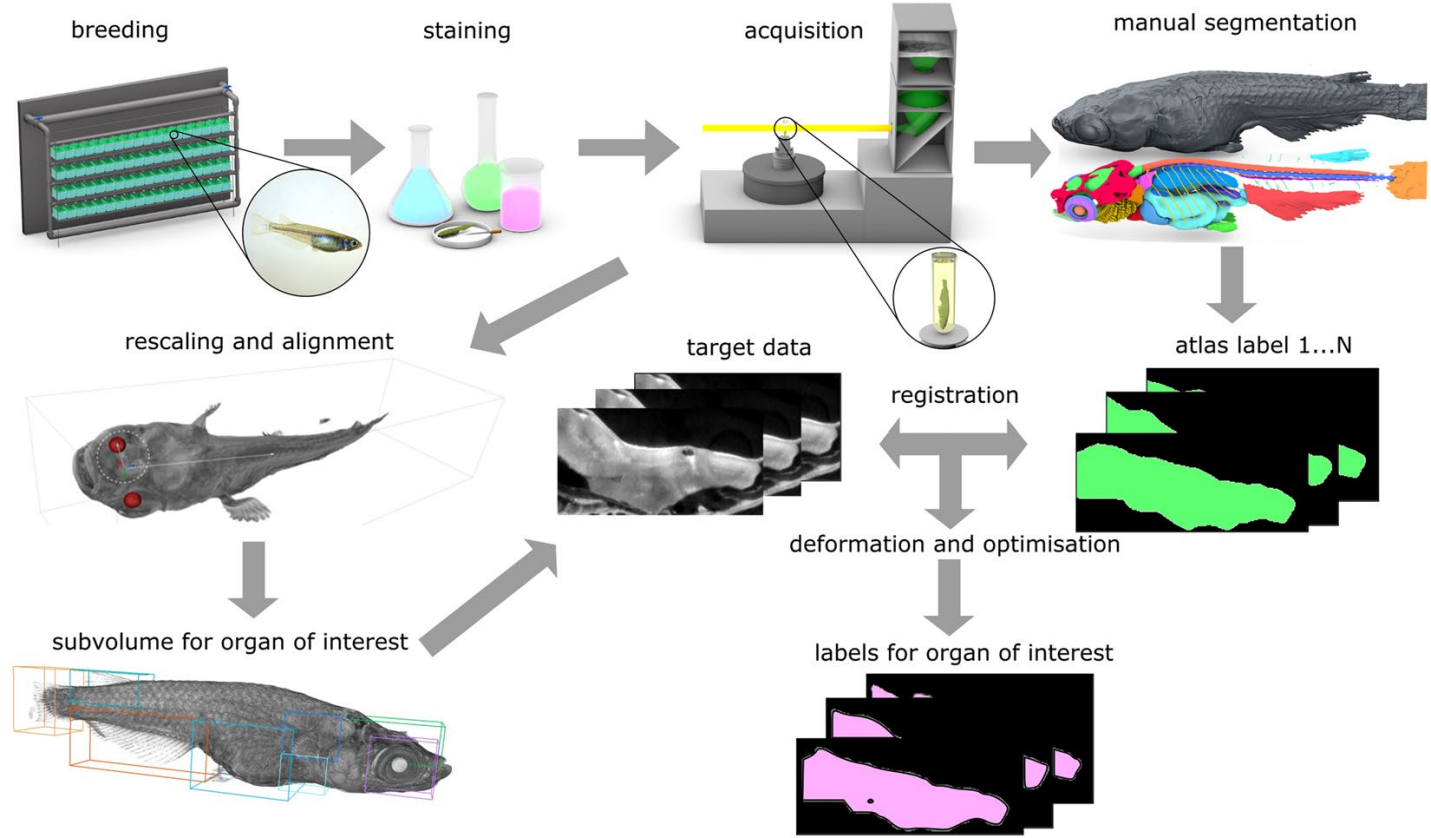
Pipeline for quantitative 3D morphometric analysis After breeding and fish husbandry, the specimens are fixed and stained with contrast agent. 3D data sets are acquired with X-ray micro-CT at the synchrotron radiation facility. Manual segmentation leads to a reference atlas. Each new dataset is rescaled and aligned with respect to the reference model. For each organ of interest the subvolume is extracted. The atlas labels for the organ of interest are then registered and deformed to create new automatically segmented labels.

The main steps of the method’s pipeline of micro-CT based morphometric analysis are illustrated in Fig. 1. Specimens are subjected to sample preparation, followed by tomographic scan acquisition and data processing for 3D tomographic image reconstruction and finally segmentation and morphometric analysis.

### Optimisation of contrast for micro-CT of teleosts

X-ray micro-CT is a valuable tool for 3D imaging for a wide range of applications. Apart from high penetration power, it offers various spatial and time resolution scales and multiple contrast modes. To visualize whole adult teleosts with sufficiently high spatial resolution and highest achievable contrast, both between and within various organs, and to enable automatic segmentation, we investigated alternative staining procedures to optimize absorption contrast. The use of contrast agents for X-ray imaging has been reported previously^13,16,17,24–26^. Published staining procedures with contrast agents are optimised for overall rather than differential contrast or specifically designed for generating contrast of only one particular organ or tissue, with special focus on embryonic or earlier developmental stages specimens.

**Figure 2:**
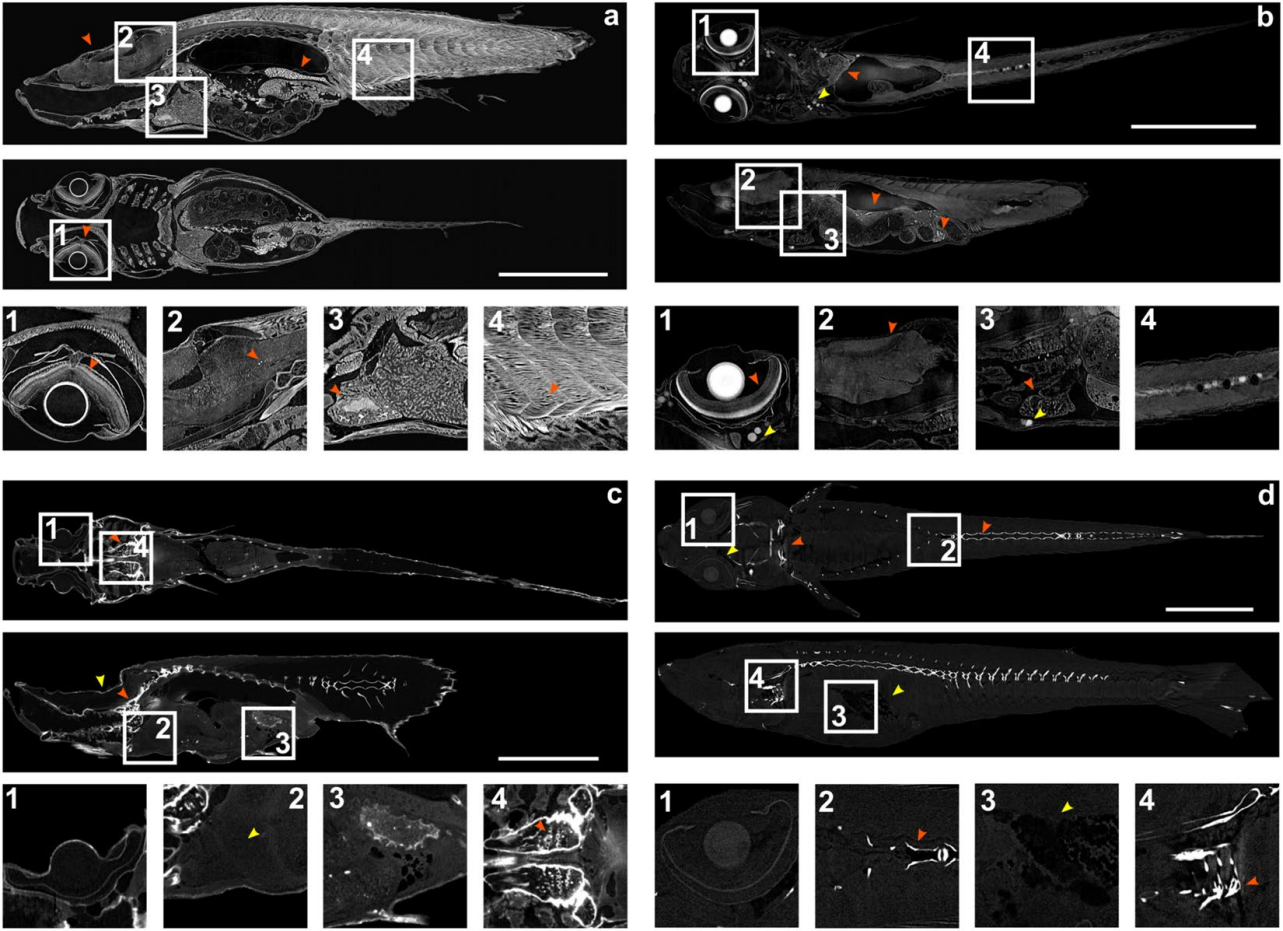
Optimisation of absorption contrast. Coronal and sagittal virtual sections of adult medaka stained with PTA (**a**), I_2_KI (**b**), EuCl_3_ (**c**) and unstained control (**d**). Enlargements of boxed regions are shown. Note the superior absorption contrast of specimen stained with PTA. (**a**) PTA staining results in good differential absorption of all body regions and tissues, including tail musculature (red arrow heads). (**b**) I_2_KI staining results in differential absorption in eye (1, red arrowhead). I_2_KI precipitates result in strong signals in the head region, intestine and spine (1, 3, yellow arrowheads; 4, spine). Absorption in inner organs is weak with low tissue specificity (2, 3, red arrowheads). (**c**) EuCl_3_ staining results in strong staining of bones (red arrowheads) and weak absorption in inner organs. Yellow arrowhead depicts morphological artefacts of the skull. Note weak absorption in soft tissue (2, yellow arrowhead). (**d**) In unstained adults, very weak absorption is visible in the eye (1) and gut (3, yellow arrowhead), whereas bones result in strong absorption (2, 4, red arrowheads). Scale bars: 6 mm.

Here we study application of staining to adult teleosts under the view point of suitability for automated segmentation and feature extraction. We chose medaka fish at 40 days post fertilization (dpf) as at this time point medaka have completed puberty, thus all organs of the adult medaka including the gonads are fully differentiated. To achieve the necessary differential contrast (i.e. tissue specific contrast to differentiate between individual organs and sub-regions within organs) and resolution we systematically tested different contrasting protocols to establish an optimal procedure for contrasting different adult tissues allowing high resolution analysis of whole adult specimens (Supplementary Table 1). For fixation, we have tested 4% paraformaldehyde (PFA), 4% PFA and 1% of glutaraldehyde and their combination with a detergent Tween-20 (3 specimens each). The 4% PFA fixation yielded the best results with respect to tissue preservation and penetration of contrast agents. As staining agents we evaluated: 1) iodine (I_2_KI), a widely used contrast agent; 2) phosphotungstic acid (PTA), typically used for soft tissue staining of larger samples and 3) europium chloride (EuCl_3_), a contrast agent for correlative imaging of magnetic resonance tomography and fluorescence microscopy^27^. Representative examples for whole adult medaka are shown (Fig. 2), where coronal and sagittal virtual sections through the centre of a 3D volume of a whole adult sample reveal remarkable differences.

Excellent contrasting and differentiation of all tissues was achieved with PTA staining, with high differential contrast between organs and tissues. Absorption of muscle fibres, gonads, heart muscles and brain are superior in terms of intensity, contrast and texture (Fig. 2a, movie 1), enabling automatic segmentation pipelines to be applied.

I_2_KI staining results in high contrast between some organs and tissues. For example, neuronal layers within the retina are clearly discernible as well as structures of the heart muscles. However, we and other groups have observed that I_2_KI has affinity to lipids, thus staining mostly fat and fatty tissues^17,28^. Resulting iodine precipitates reduce contrast density in other tissues and decrease the dynamic range for tissue analysis. I_2_KI precipitates are clearly visible with X-ray tomography in the head region, abdominal part and along the spine (Fig. 2b).

Europium chloride (EuCl_3_) staining results in weak absorption in soft tissues and high absorption in calcified regions. Although overall soft tissue contrast is increased compared to unstained control samples, the difference between specific tissue types and organs is low. Furthermore, EuCl_3_ appears to replace calcium in the specimen^29,30^ leading to strongly enhanced contrast of the skeleton and fragility of bone structures and resulting morphological artefacts (Fig. 2c).

The control, unstained specimen displays scarce differential absorption contrast of soft tissues. Only calcified regions of the skeleton, particularly spinal column, teeth and gills cartilage show robust absorption. In addition, fatty tissues close to the eye and liver are discernible due to lower X-ray absorption coefficient in comparison to the surrounding tissue (Fig. 2d).

**Figure 3:**
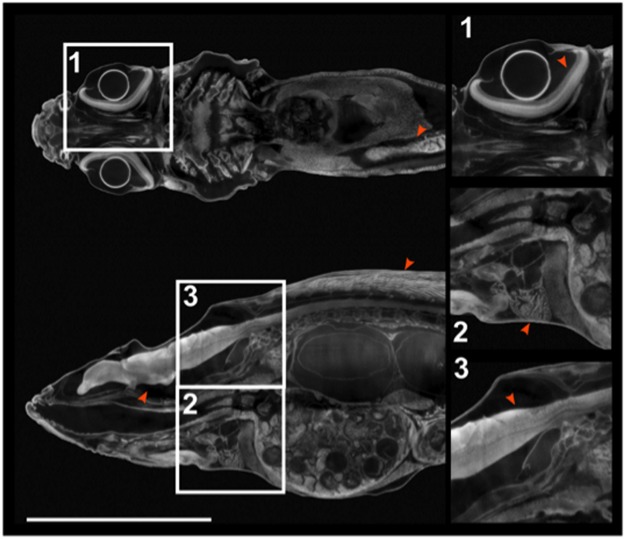
Micro CT of a juvenile zebrafish. Coronal and sagittal virtual sections of a 38 dpf zebrafish stained with PTA. Boxed regions are shown in higher magnification. As in adult medaka, all tissues and body regions show high and differential contrast: (**1**) eye, note distinct layers of neural retina (red arrowhead); (**2**) heart (red arrowhead); (**3**) brain (red arrowhead). Scale bar: 6 mm.

We therefore chose PTA as contrast agent for adult medaka imaging for its high, well-balanced differential contrast for all tissues. X-ray imaging of PTA stained adult zebrafish showed equally superior results with respect to intensity, contrast and texture (Fig. 3). The results demonstrate that PTA provides optimal contrast for X-ray imaging of these adult teleosts.

3D rendering of the PTA-stained volume reveals high structural information, such as fin rays, scales and cranial lateral line neuromasts (Supplementary Fig. S1b). Three-dimensional rendering of an entire adult medaka shows high differential contrast and the high spatial resolution allows to discern subtle details and different structures within organs. As examples, we show 3D visualisations of brain, eye and intestine. 3D rendering of the brain reveals the overall tissue morphology (Supplementary Fig. S1a). In the adult eye, the individual nuclear layers of the neural retina are discernible by 3D rendering. Optic nerve, lens and associated tissues, such as choroid and cornea manifest high differential absorption contrast ((Supplementary Fig. S1c). The overall morphology of the medaka gut, including the intestinal folds along the rostro-caudal axis of the intestinal tract is clearly resolved (Supplementary Fig. S1d, movie 1).

In summary, we identified and incorporated a staining protocol into our pipeline, which results in high differential absorption contrast in soft tissues of adult specimens and the spatial resolution required for automated segmentation, in our particular case <5 µm. Importantly the staining method provides a fast, simple and affordable sample preparation based on easily accessible reagents. It overcomes problems resulting from tissue size and opacity, which are frequently an issue with adult samples^25^. While some optimisation of staining protocols, for example incubation time, might be required for other species, the main steps are thus applicable for X-ray imaging of a wide range of small vertebrates.

### 3D atlas of adult medaka as interactive reference model of medaka anatomy

Micro-CT imaging based on our protocol provides the complete 3D anatomical information of the whole individual. The high quality regarding differential contrast, signal to noise ratio and resolution predestine our dataset for morphological segmentation of the whole body. A segmented 3D data set exploits the inherent advantages of micro-CT as a quantitative method and delivers a valuable resource for the scientific community as it provides the morphometric information in an accessible, user friendly format.

**Figure 4:**
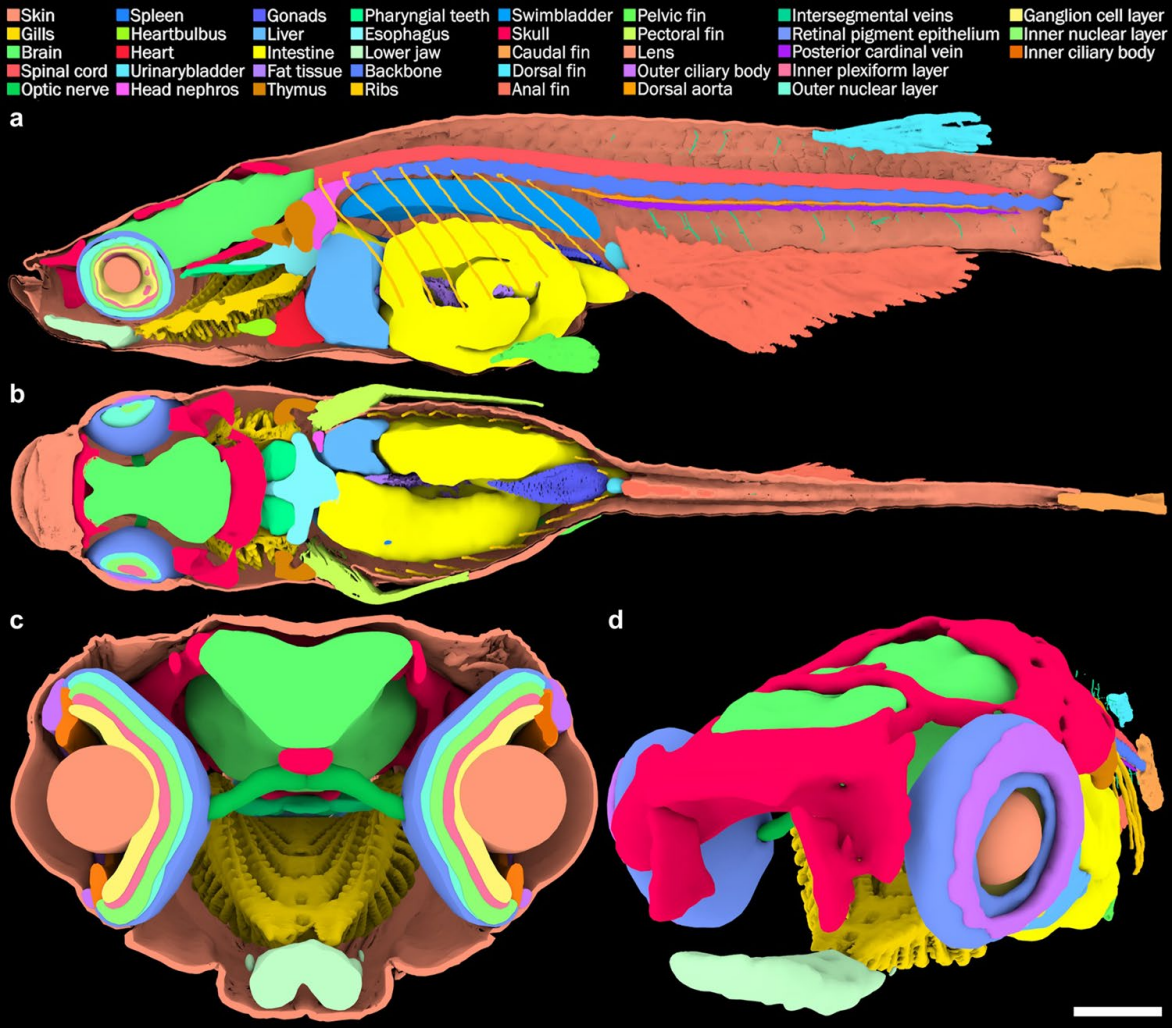
3D anatomical atlas (**a**) sagittal, (**b**) coronal, (**c**) transverse planes through the 3D interactive model. Each colour represents a unique anatomical organ. Muscles are not shown for better visibility of other organs. (**d**) 3D view of the model (skin is rendered transparent).

We therefore established an atlas of an adult female medaka based on manually segmented 3D data, that is accessible as an interactive 3D PDF document (Fig. 4, Supplementary Fig. S2). For this reference atlas, the micro-CT data were first manually segmented at each 20^th^ of 3140 virtual sections in Amira (version 5.4.2; FEI Visualization Sciences Group). The organs were identified based on histological atlases available for teleosts^31,32^. For the manual segmentation of the anatomical model, smooth and coherent structures were annotated, resulting in a 3D anatomical atlas (movie 2). Exploiting the high micro-CT data quality, we further improved the atlas by 3D semi-automatic segmentation of the individual organs applying a random walk algorithm implemented in the Biomedisa application^33^. The individual organs were then embedded in an interactive 3D PDF document as previously described^34^. The resulting atlas allows to rotate, translate and zoom-in on the feature of interest.

The final interactive 3D medaka anatomical atlas contains 38 annotated organs (Fig. 4, movie 3). To simplify visualisation, we subdivided the atlas into different groups such as skeleton, muscles, skin, eyes, as well as respiratory, cardiovascular, urogenital, immune, nervous and digestive system respectively. Each system can be distinguished from the surrounding tissues by means of transparency settings (opaque, semi-transparent and transparent depicted as an eye symbol). The organ of interest can be further virtually dissected by means of the isolation tool (target sign located on the right side of the transparency settings). View angle and zoom factor can easily be adjusted without additional software. The measurement tool (3D Measurement Tool in Adobe Acrobat) provides users with information on the physical dimensions of the structure of interest in millimetres (movie 4).

The interactive PDF file of the 3D virtual atlas (Supplementary Fig. S2) provides a tool for an interactive study of medaka anatomy with the option for further analysis of anatomical details. The user-friendly access to 3D quantitative information allows unprecedented virtual dissection of external and internal features without the need to scan through large sets of data. Similarly, the methods pipeline can be employed to establish 3D atlases in other models of interest.

### Comparative morphological analysis based on organ-specific automated segmentation

The digital morphological atlas of the adult medaka fish enables a quantitative morphometric analysis of all segmented internal and external features, including organs and tissues. Therefore, the atlas is to be taken as a reference model for comparative studies relating to phenotypic analysis and is thus of crucial importance for the further use of medaka as a model organism.

The segmented data sets contain information of relative position, shape and size of each segmented organ in relation to the body. Such quantitative description can, in principle, be used in comparative approaches to search for morphometric variance between individuals. However, the analysis of larger specimen numbers is impeded by the very time-consuming semi-manual segmentation.

Therefore, we used the reference atlas to develop an automated segmentation procedure for high-throughput comparative studies. Automated segmentation consists of three steps**:** (1) alignment and scaling of datasets to the reference atlas at the level of the entire body (whole body binary mask); (2) application of a set of organ specific procedures tailored for automatic segmentation of the respective organs in a sub volume determined by a corresponding bounding box; (3) assembly of the segmented organs, reconstituting the individual.

The organ specific procedures employ organ specific position, shape and differential contrast properties. This allows to carefully segment each organ individually based on specifically adapted algorithms. For example, for automatic segmentation of the eyes and spinal cord the expected shape of the resulting label (number allocated for each voxel specific for the tissue of interest) was taken into account, namely a sphere and a tube respectively^35–37^. Segmentation of organs such as head nephros and liver was based on the texture analysis due to high tissue contrast^38–40^. To date, we have successfully employed automatic segmentation for eyes, liver, head nephros, heart, spinal cord, intestine, spleen, and brain. The segmentation also yields quantitative morphometric parameters, such as total volume, surface area and center of mass (position) for each anatomical feature and/or organ. These parameters allow quantitative comparison of the segmented organs. Depending on the size of the volume, the average time for automatic segmentation of one organ takes about 20 minutes. In comparison to manual segmentation (2 hours per organ), it is 6 times faster and unbiased. Provided that similar machine power is used (see methods chapter), the reconstruction and automatic segmentation times will be similar. The fidelity of automatic segmentation is comparable to manual segmentation by more than 90% (Supplementary Fig. S4).

**Figure 5:**
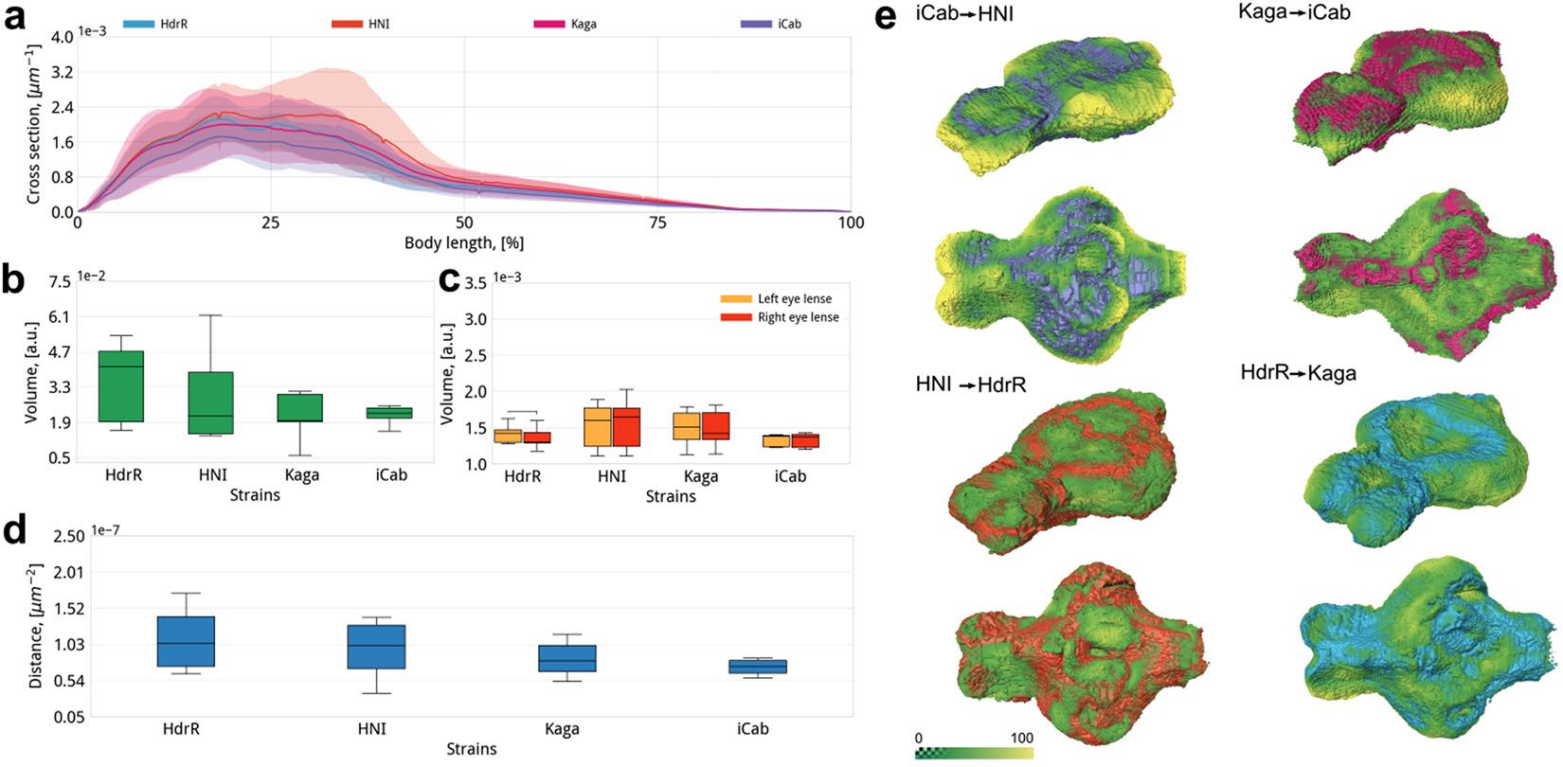
Comparative morphometric analysis (**a**) Comparison of cross sectional areas along the antero-posterior axis (position indicated as percent of the total length); the head region comprises the anterior 30% of the total length. Solid lines are the average area of the cross section per strain, the semi-transparent region shows the standard deviation. All parameters are normalized to the total volume of specimens. Cross sections of iCab head region are smaller than those of HNI, HdrR and Kaga. (**b–e**) Comparative analysis of quantitative morphological parameters between the four isogenic inbred strains. Number of specimen is 7, 8, 5 and 6 for HdrR, Kaga, iCab and HNI respectively. (**b**) Comparison of brain volume, which is smaller in Kaga and iCab than in HdrR. (**c**) Comparison of lens volumes, showing that iCab and HdrR lenses are smaller than those of HNI and Kaga (**d**) Comparison of the distance between the eyes, which is bigger in HdrR compared to iCab and Kaga (**e**) Comparison of 3D strain specific brain shape morphometrics, shown in violet, magenta, orange and cyan for iCab, Kaga, HNI and HdrR strains respectively; antero-lateral and ventral view of brains. The strain specific shape is overlaid with the displacement distances (color-coded: green → yellow) to indicate variation in micrometers for each strain to be transformed into another one (for example, iCab → HNI). Note that different transformation patterns emerge from the pairwise comparisons.

In the literature, specific morphometric differences of external morphology as well as inner organs have been identified between established isogenic inbred medaka strains^41,42^. To validate the analytical power of our image acquisition and analysis pipeline we tested whether it uncovers strain specific morphometric features. We therefore analysed samples of four isogenic inbred medaka strains (iCab, HdrR, HNI, Kaga) and subjected these to the automated comparative morphometric analysis. We first analysed the overall shape of five individual fish per isogenic strain by evaluating the area of cross sections along the antero-posterior axis as shown in Fig. 5a. The area of individual cross sections was used for a quantitative comparison between the four isogenic strains. We found that the resulting shape profiles of this cross section area contained unambiguous strain specific features. Namely, a morphometric comparison of these shape profiles revealed that iCab specimens are characterized by a 20% smaller head region (anterior 30% of the total length) compared to HdrR, HNI and Kaga.

Strain specific brain morphologies and sizes have been reported in a comparative analysis of different inbred medaka strains^41^. We therefore compared brains from the isogenic strains by automatic segmentation and morphometric analysis. Automatic segmentation yielded results comparable to manual segmentation of the brain (Supplementary Fig. S3a). An example of automatically segmented brains of eight randomly chosen specimens among samples of four inbred strains is shown. With respect to a manual dataset, automatic segmentation yields comparable results concerning segmentation quality (Supplementary Fig. S3a). All automatically segmented brains share a typical overall shape, with clearly discernible telencephalon, hypothalamus, optic tectum, cerebellum and hindbrain^31^. With limited user intervention, minor segmentation errors at the brain-skull interfaces can be corrected.

To enable comparative analysis of quantitative parameters in an organism with life-long growth, extracted organ specific parameters were normalized to the total volume of each specimen, thus resulting in a quantification independent of absolute body size. The comparison of brain and lens volumes for different inbred strains revealed strain specific differences of shape and size (Fig. 5b–d). Volumetric comparison showed that the brain volume of iCab and Kaga is smaller than that of the HdrR strain (Fig. 5b). The lens volumes of iCab and HdrR are smaller than those of HNI and Kaga (Fig. 5c). Also, the distance between the eyes of Kaga and iCab is smaller than in HdrR (Fig. 5d).

To fully explore the micro-CT data, we compared strain specific shape morphometrics of brains. Multiple automatically segmented brain labels (voxels of a specific organ) were compiled into a 3D probability map for each strain (Supplementary Fig. S3b). Based on the probability map, the 3D brain shape morphometrics for each specific strain were selected (>60% probability in the compilation). The 3D strain specific shape morphometrics (depicted in violet, magenta, orange and cyan colors for iCab, Kaga, HNI and HdrR respectively) were then used to calculate displacement fields between brains of different inbred strains. Such distance maps allow to quantify specific morphological differences not revealed by other approaches (i.e. brain volume, Fig. 5b) and reveal where brain morphology varies in a strain specific, quantitative manner. This morphometric comparison showed the highest shape divergence (up to 100 µm distance) between iCab and HNI (Fig. 5e). When compared to HNI, iCab brains have a shorter telencephalic region and a narrower optic tectum. The telencephalic portion is more similar in shape when iCab and Kaga are compared, whereas the iCab optic tectum is wider than that of Kaga. HNI and HdrR on the other hand share more similar overall brain shapes. Also, HdrR and Kaga share more overall shape similarity. To summarize, our morphometric analysis uncovers that the iCab brain is morphologically most distinct when compared to the other isogenic inbred strains with a short telencephalic region and a narrow optic tectum of the dorsal midbrain.

Thus, our comparative morphometric analysis detects quantitative differences not only of external body morphology but importantly also of shape and size of inner organs. In good agreement with previous reports^41,42^ we detect strain specific morphometric variance of the general head morphology but also in organs such as the brain and eyes. Using automated segmentation thus allows to compare large numbers of specimens in a quantitative manner, which is the basis for future genetic studies.

## Discussion

Quantitative morphometric analysis using state-of-the-art multidisciplinary bio-imaging approaches is essential to identify genotype-phenotype associations. Due to the high penetration power, X-ray tomography is widely applicable to diverse specimens at various resolution scales and fast scanning time^43^. Chemical contrast agents increase X-ray absorption of biological soft tissues and can thereby aid to visualize the tissue of interest^44^. Importantly, affordable laboratory X-ray micro-CT scanners and table top synchrotron sources make X-ray absorption tomography easily applicable for many scientist^45^. In this study, our aim was to use micro-CT for high resolution imaging of the entire adult small vertebrate with all tissues to obtain a complete 3D description for quantitative anatomical analysis.

We implemented a complete pipeline consisting of sample preparation and mounting, image acquisition, 3D rendering of the data and importantly segmentation of the 3D data to create a user friendly and versatile interface for visualization and analysis. We tested our pipeline on widely used genetic teleost models, such as medaka and zebrafish^2,46,47^. Among tested contrast agents, PTA provided the best differential contrast in all tissues without visible morphological artefacts or formation of precipitates. The high differential contrast of PTA in combination with high spatial resolution of X-ray micro-CT imaging allowed whole body 3D imaging with discrimination of organs and tissues of adult medaka and zebrafish.

High resolution X-ray micro-CT provides a precise quantitative volumetric data set. Due to the size and complexity of the adult vertebrate organism this data set is large and inconvenient to analyse by biologically oriented users. We therefore converted our data by manual segmentation into a user-friendly interactive 3D morphological atlas comprising the quantitative description of the adult body with 38 annotated organs. It is anticipated that the 3D anatomical atlas will evolve through contributions from the medaka scientific community. It is thus conceivable to introduce segmentation of organs into different regions to add a new level of resolution to our atlas. To further exploit the quantitative nature of micro-CT data, we developed organ-specific automated segmentation algorithms for the morphometric analysis of large specimen numbers.

Digitalized atlases have proven successful for automatic segmentation of various organs, such as the cerebral anatomy using MRI data^48^. X-ray micro-CT has been previously used for 3D imaging of mice, *Xenopus* and zebrafish^17,44,49,50^. While these studies have demonstrated the power of X-ray imaging, in most cases the downstream analysis in terms of digital atlas, segmentation and morphometric analysis was not exploited with the exception of Wong *et al*., who reported a morphometric CT based analysis of mouse mutant embryos^22^. Semi-automated segmentation of vertebrae has been used for a comparative analysis of mutant and wild type zebrafish^51^. Since our aim was to employ micro-CT data for comparative morphometric analysis of large sample sizes we implemented automated segmentation as a user friendly and fast image analysis.

Here, to minimize computation time without compromising on the spatial resolution, we used our 3D interactive atlas as a reference model in a pyramid approach at different resolution scales. Each of the 38 organs, tissues and structures of interest can subsequently be segmented individually with an optimised segmentation algorithm depending on contrast, texture and 3D shape. As a future development this approach can be customized for other applications in form of a user toolbox. While the reference atlas and dataset are provided by the user, the suitable optimised segmentation algorithm is automatically chosen and adapted based on the image quality parameters. Thus, our pipeline is highly versatile and readily adaptable to the requirements and features of other model systems.

Previous reports demonstrated heritability of morphometric variance between isogenic medaka strains, hinting at a wealth of yet unexplored genotype-phenotype associations of complex genetic traits^47^. We demonstrate that our pipeline readily uncovers craniofacial variance of medaka inbred strains. This is in good agreement with previous studies addressing craniofacial traits in medaka^42^. Analysis of craniofacial variance relies often on parameters of outer head morphology. Micro-CT based imaging provides precise morphometric information of the entire body, including inner organs. By automated segmentation we therefore extracted morphometric parameters of inner organs and used them in a comparative analysis. Importantly, in addition to gross morphometric parameters such as surface area and volume of a given organ, we have successfully analysed more subtle shape related parameters in our comparative morphometric analysis. We find strain specific shape variance between brains of specific isogenic medaka strains. Strain specific variance of brain morphometrics has previously been shown using conventional organ dissection and light-microscopy^41^. The 3D displacement map thus serves as a means to detect phenotypic shape differences of the body as well as selected tissues and organs.

Our micro-CT based approach in combination with automated segmentation provides several advantages. Firstly, the entire body is imaged and thus all organs and tissues that are resolvable by segmentation can be analysed. Secondly, our automated segmentation pipeline reduces the computational load, thus enabling to process and analyse large data sets in a standard lab environment. This is of crucial importance for genetic studies that rely on statistical approaches to uncover genotype–phenotype associations, such as genome wide association studies (GWAS). Thus, our digital atlas in combination with automated segmentation of specimens paves the way for state-of-the-art genomic approaches to unravel the genetics underlying variance of quantitative traits for all features that are included in the atlas. With the upcoming developments in online reconstruction and image processing algorithms, monitoring and controlling efficiency of imaging experiments, the 3D X-ray micro-CT will allow upscaling to process large numbers of individuals to study the genetic basis of such quantitative morphological traits. Furthermore, small vertebrate models such as medaka are increasingly used as disease models to study how disease affects organ function, which is often caused by altered organ morphology^3–5^. Thus, micro-CT will provide important morphometric information to study subtle disease phenotypes that are difficult to assess otherwise and by that provide information on disease etiology.

## Methods

### Fish stocks and husbandry

Medaka (*Oryzias latipes*) stocks and zebrafish (*Danio rerio*) stocks were maintained at the Institute of Toxicology and Genetics (ITG) of the Karlsruhe Institute of Technology (KIT) as described previously in recirculatory systems under 14 h light/10 hours dark conditions at 26 °C^52^. The following isogenic inbred strains medaka strains were used: HNI-II, HdrR-II1, iCab and Kaga. Fish were raised at low density (1 fish per litre), to avoid stress related variation of development. Animal husbandry and experimental procedures were performed in accordance with German animal protection regulations (Regierungspräsidium Karlsruhe, Germany; Tierschutzgesetz 111, Abs. 1, Nr. 1, AZ35-9185.64/BH). The facility is under the supervision of the Regierungspräsidium Karlsruhe who approved the experimental procedures.

### Micro-computed tomography

#### Data generation pipeline

A flowchart of the method’s pipeline is depicted in Fig. 1. For the studies reported here, we chose the teleosts zebrafish (*Danio rerio*) and medaka (*Oryzias latipes*). To improve contrast, we optimized the sample preparation protocol with respect to fixation, staining and embedding. About 2000 X-ray projection images per tomogram were acquired at the high resolution tomography end station of the synchrotron light source facility at KIT^53,54^. To avoid deterioration of specimens only freshly fixed and stained samples were used and storage of samples was avoided. We found that X-ray absorption contrast of similar tissues between different samples is statistically the same (typical Z-test value of 0.25). Thus, our preparation protocols and acquisition parameters guarantee reproducible spatial resolution, contrast and quality of 3D datasets. The 3D volumes were reconstructed with the help of a GPU-based fast image processing framework with the typical 3D reconstruction time of 5 minutes^55,56^. Depending on the organ, anatomical features were manually segmented on one specimen with low precision for each twentieth slice of the 3D volume. The average time for manual segmentation was about 1.5–2 hours per organ, thus for an entire specimen it took approximately 76 hours to complete. To increase accuracy of segmentation we used the biomedical image segmentation application^33^, creating thus a high-resolution 3D model of the adult medaka fish anatomy. If the semiautomatic segmentation failed, the slice of the highest error was segmented manually, and the semiautomatic segmentation was repeated. The manually segmented labels were validated and annotated by biologists. 3D volumes of other samples were automatically segmented based on the initial anatomical atlas. The automatic segmentation was parallelized over 30 CPUs and took 20 min per organ (depending on the size).

#### Sample preparation

Sexually mature medaka fish (8 weeks post hatching) and 38 dpf (days post fertilization) zebrafish were sacrificed and fixed using 4% formaldehyde/PBS with 1% glutaraldehyde respectively. To improve X-ray absorption of soft tissues, fish (iCab strain) were stained with 0.1% iodine potassium iodide (Lugol’s solution, 25 specimens), 1% europium chloride (EuCl_3_, 12 specimens) or 0.3% phosphotungstic acid in 70% ethanol respectively (15 specimens). If not stated otherwise, fish were fixed for three days at room temperature followed by staining with contrast agent for three days at room temperature. The solutions were changed every 48 hours. Based on our experience shorter incubation times are not sufficient for a whole-body penetration of adult fish. After the staining procedure, samples were washed in PBS, embedded in 4% agarose and sealed in polypropylene containers. All sample preparation procedures were performed in a fume hood with appropriate safety protection.

#### Imaging

All specimens were imaged at the tomographic imaging station at the synchrotron radiation facility of KIT. From a white beam of a bending magnet (critical energy of 6.1 keV), 16 keV X-rays were selected by the Double-Multilayer Monochromator with 2% energy bandwidth with an additional Al filter (0.2 mm) to minimize radiation dose. X-ray projections were detected by a CMOS camera (pco.edge, 2560 × 2160 pixels, 6.5 × 6.5 µm² pixel size) coupled with an optical light microscope (total magnification 3.6×). Photons were converted to the visible light spectrum by a LAG scintillator of 50 µm. A complete optical system resulted in an effective pixel size of 1.81 µm and the measured spatial resolution of 5 µm. For each specimen, a set of 2000 projections with 400 ms exposure time each were recorded over a full 360° tomographic rotation axis. With the extra time required for reference images and mechanics, the total scan per tomogram was 20 minutes. As the field of view was only 4.6 mm × 3.9 mm, to cover the entire length of each sample (approximately 2 cm), the sample was vertically translated after each tomogram. Thus, a set of (typically 5) tomography acquisitions per specimen was acquired.

#### Reconstructions

The projections were pre-processed for dark noise of the camera (including not active pixels of the sensor), reference beam and inhomogeneities of the scintillator (bright scintillating spots) in MATLAB. A set of projections were reconstructed to a complete 3D tomographic volume using the GPU-based PyHST algorithm^56^. 3D volumes of each specimen were correlated and combined into a single 3D volume in MATLAB and then further denoised by non-local means filter. The machine used for 3D reconstructions had openSUSE 13.1 as an operation system, RAM 258 GB, GPU: 6x GeForce GTX TITAN and 2xIntel(R) Xeon(R) CPU E5-2680 v2 @ 2.80 GHz (20 threads per CPU).

### Volume analysis

#### Segmentation

As a basis of the 3D anatomical atlas, the X-ray tomography dataset of an adult female medaka fish was manually segmented using Amira software based on the already available atlases and annotations^31,32,57,58^. The resulting manually segmented 2D slices were used to label remaining slices by applying the semiautomatic segmentation application Biomedisa^33^. This procedure is similar to interpolation between the slices but leads to much better results in terms of precision, smoothness of the labels and fine feature detections, for example muscles. Segmented organs were visually checked by group of biologists. Each segmented individual organ was converted to the surface by SurfaceGen module. The smoothing property was set to ‘unconstrained smoothing’, also the options ‘add border’ and ‘adjust coords’ were activated. Then the Simplifier tool was selected with the final number of faces comprising the model set to the value which allow to preserve the structure of the model, while reducing the number of faces. To the simplified model, the SmoothSurface module was connected to reduce remaining sharp corners by optimization of the vertices position. The number of iterations and the lambda smoothing parameter were set to 2 and 0.6 correspondingly, for all individual surfaces. The final smoothed components were exported as Wavefront (*.obj). Afterwards, each component was opened in a common project of Cinema 4D (versions 12 & 14; Maxon Computer GmbH). The tree of the project served as a hierarchical model of the atlas, where each object was placed according to its relation to the system. For each individual component, Polygon Reduction object was added to perform more exhaustive simplification. The co-planar optimization and the boundary curve preservation options were set to active, and the mesh quality factor parameters were assigned to 1000. The reduction strength parameter was set for each model individually, resulting in reduction strength of 70–90% with respect to initially segmented labels. The simplified model was then exported to COLLADA 1.4 (*.dae) format and opened in Deep Exploration (Version 6; Right Hemisphere®). Assignment of the colors to each individual organ was done in the Material (Scene) panel. Small and incoherent structures were not annotated and are not displayed in the atlas. The final model then was exported as a Universal 3D (*.u3d) data format. Details on polygon optimization and embedding in a 3D PDF files have been published previously^34^. The model was then opened with Adobe Acrobat 10.1.1. The interface to interact with the atlas was created by use of embedded functions in Tool pane, where the action to each button was assigned by JavaScripts in JSON format.

#### Automated segmentation

Automatic segmentation of specimens relies on the 3D atlas (reference dataset) described above and consists of the following steps (see Fig. 1): (1) rescaling and alignment of 3D volumes and subsequent segmentation with respect to the coordinates of the reference dataset; (2) definition of the 3D sub-volume within which all specimen points lie, so called bounding box for the organ to be segmented; (3) registration of the features within the bounding box of the organ to the reference mask; (4) elastic deformation of the reference mask on the segmented organ; (5) optimization of the obtained mask.

The 3D volumes were first aligned based on a three-point calculation: both eyes (identified by sphericity) and tail (posterior 30% of total body length).

The segmentation of the eyes was performed based on the assumption that the lens of the eye is a spherical object and that each specimen should have two lenses. Since eye lenses are typically surrounded by water, all area within the specimen volume was calculated based on Otsu thresholding. Based on the connected components analysis all unconnected water regions were allocated a specific label (number allocated for each voxel specific for the tissue of interest). To find the lens, we first calculated the volume and sphericity for each discrete labeled region and then composed a self-similarity matrix for differences in volume. Two labels with high sphericity and self-similarity values were denoted as lenses.

The whole-body binary mask of a specimen was created based on the Otsu thresholding followed by morphological closing operations^59^. The resulting 3D volume mask was aligned with respect to the reference atlas by means of a low-pass pyramid representation. The initial (to be segmented and reference) datasets were reduced by a factor of 12, then aligned sequentially with rigid, affine and diffeomorphic transformation (epsilon value to stop optimization procedure was chosen to be 1e-6). The volumes were up-scaled (8 times reduction compared to the original dataset) and the transformation matrix refined. The steps were repeated for scaling factors 8, 4 and 2. At each scaling level, 1000, 500, 250 and 0 iterations were performed with 4, 3, 2 and 1 sigma voxels Gaussian smoothing. This multi-scale approach guarantees high precision of the transformation operations required with high resolution data. At the same time, it minimizes the computational time, since only fine adjustments of transformation operations are required. The result is an aligned 3D volume mask and a matrix of transformation parameters.

These transformation parameters were used to deform organ-specific labels (eye, brain or others) from the reference atlas and to select a sub-volume (bounding box compromising the complete label) corresponding to the organ of interest. From this step on, each label was registered (transformation of reference mask into the organ of interest label) separately in the corresponding sub-volume. Similar as in an entire fish, the registration procedure is repeated with the low-pass pyramid representation of a sub-volume. Rigid and affine registrations were performed with 4 scaling factors (12, 8, 4, and 2) with 1000, 500, 250 and 100 iterations and 4, 3, 2, and 1 voxels as sigma for Gaussian smoothing. Final diffeomorphic (smooth continuous mapping) registration was performed at 5 scaling factors (10, 6, 4, 2 and 1) with 500, 500, 500 and 250 iterations each and 5, 3, 2, 1 and 0 voxels as sigma for Gaussian smoothing. All registration operations were performed based on the Toolkit registration framework^60^.

Inadequate and abnormal datasets were excluded before segmentation, based on the whole fish mask (for example abnormalities in the histogram distribution or if samples were larger than the imageable area resulting in incomplete data sets) and histogram distributions of a reconstructed 3D volume, excluding thus artifacts (if occurred) at sample preparation or imaging steps. All scripts for automatic segmentation were written in Python programming language with the use of ANTs software to perform elastic registration.

To evaluate the fidelity of the automatic segmentation, we have compared the similarity of manual and automatic segmentation by calculating the dice similarity coefficient. Three brains per each strain were randomly chosen and segmented manually. The similarity of these manually segmented labels with results of automatic segmentation is shown in Supplementary Fig. S4. Most of the labels have a similarity to manual segmentation of more than 90%. The same computer as for reconstruction has been used for automatic segmentation using all both CPUs and all 20 cores (each). The time for automatic segmentation may vary depending on machine power.

#### Morphometric analysis

For the comparative morphometric analysis a total of 20 individuals (5 for each strain) from iCab, Kaga, HNI and HdrR were used. Parameters extracted by the automatic segmentation were used for the quantitative morphometric analysis of total volume (µm^3^), surface area (µm^2^), width (µm), height (µm) and length (µm) in the reference coordinate system. The centre of mass for each label was used to calculate the distance between the eyes. To describe the overall shape of the inbred strains, we employed cross-sectional analysis (area and circularity ratio) along the antero-posterior axis.

All morphometric parameters are normalized to the total volume of the specimen except an established length-thickness relationship, and thus are independent of the absolute size and its variation between the specimens.

For the morphometric shape analysis of brains, the automatically segmented brain labels (for each strain independently) were aligned with respect to the antero-posterior axis and centre of mass (see Supplementary Fig. S3). The aligned brain labels were averaged in 3D thus resulting in the 3D probability map of brain shape. This probability map allows to predict a strain specific 3D brain volume for further experiments.The strain-specific shape morphometrics was then defined by thresholding of 60% and higher probability (region within magenta line in Supplementary Fig. S3). To quantify phenotype differences, first brain morphometrics were aligned with respect to each other (antero-posterior axis and centre of mass) and then a distance map between the two surfaces was calculated. All operations were performed in Amira 6.0.

#### Visualisation

For visualisation of the 3D data we used the software Amira 6.0. Main and Supplementary Figures were prepared with the help of Inkscape 0.91 and Adobe Photoshop. All 3D renderings and video files were generated on Windows 7 as an operational system, CPU Intel® Core™ i7-3930K, RAM 64 GB and GPU: NVIDIA GeForce GTX 680.

## Electronic supplementary material

Dataset 1

Movie 1

Movie 2

Movie 3

Movie 4

## Data Availability

The datasets which support the findings of this study are available in the *figshare* repository. The access link will be provided by the corresponding authors upon request. All codes used in the current study are available on GitHub repository (https://github.com/AFSRepo/AFS-Segmentation) as open access.
