# Supplementary material for: Quantitative morphometric analysis of adult teleost fish by X-ray computed tomography
Source: Sci Rep. 2018 Nov 8;8:16531. doi: 10.1038/s41598-018-34848-z (PMC6224569; doi:10.1038/s41598-018-34848-z)
Supplement: Supplementary file 1 — Dataset 1 [file 41598_2018_34848_MOESM1_ESM.pdf]

Supplementary Information

Quantitative morphometric analysis of adult teleost fish by X-ray computed tomography

Venera Weinhardt[1,2,3], Roman Shkarin[3,4], Tobias Wernet[2,5*], Joachim Wittbrodt[2], Tilo Baumbach[1,3] and Felix Loosli[5]

[1]Institute for Photon Science and Synchrotron Radiation, Karlsruhe Institute of Technology, Hermann-von-Helmholtz-Platz 1, Eggenstein-Leopoldshafen, Germany

[2]Centre for Organismal Studies, COS, Heidelberg University, Im Neunheimer Feld 230, Heidelberg, Germany

[3]Laboratory for Applications of Synchrotron Radiation, Karlsruhe Institute of Technology, Karlsruhe, Germany

[4]Institute for Applied Computer Science, Karlsruhe Institute of Technology, Karlsruhe, Germany

[5]Institute of Toxicology and Genetics, Karlsruhe Institute of Technology, Karlsruhe, Germany

*current address: Center for Biological Systems Analysis, Freiburg, Germany

Correspondence should be addressed to V.W. (venera.weinhardt@kit.edu), J.W. (jochen.wittbrodt@cos.uni-heidelberg.de) and F.L. (felix.loosli@kit.edu)

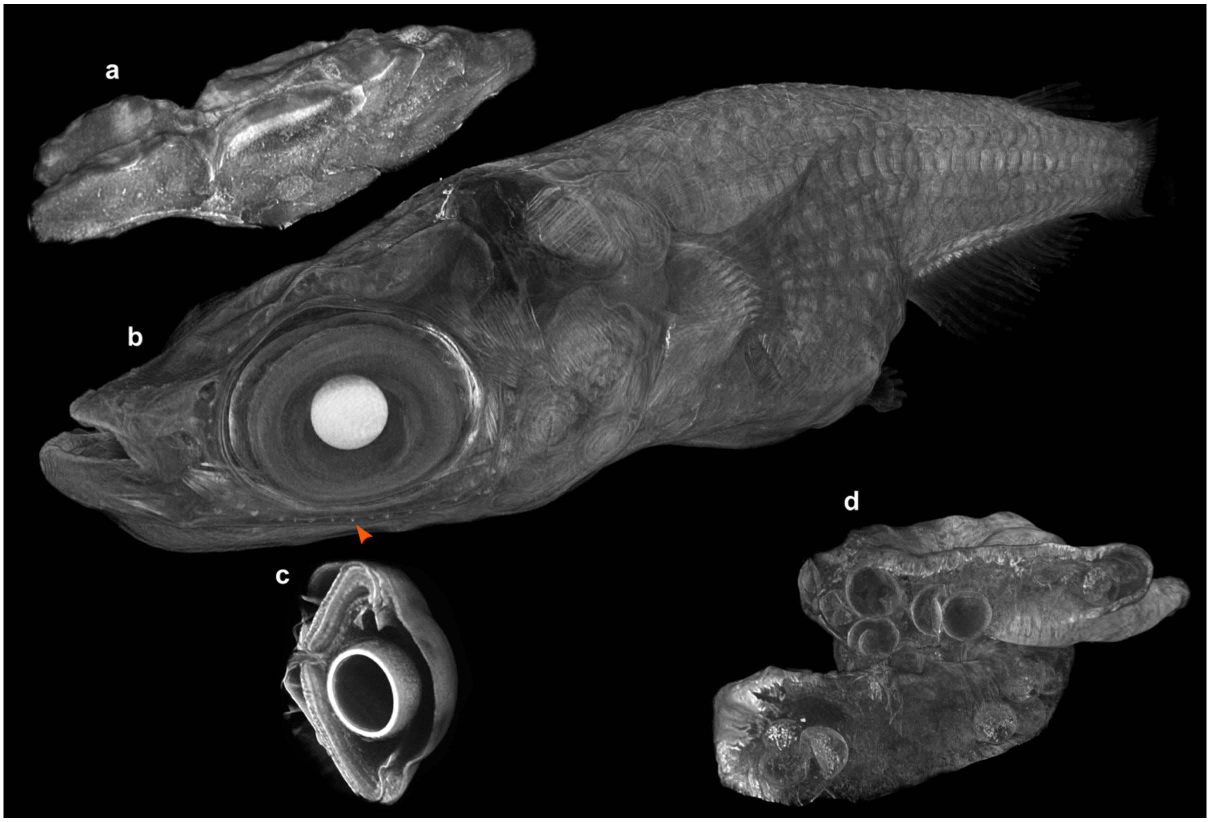

Supplementary Figure 1 3D rendering of the PTA stained adult fish

(**a**) 3D rendering of the brain shown at 5 x magnification. (**b**) 3D rendering of an entire adult medaka fish, length is approximately 22 mm. Red arrowhead indicates neuromast of the cranial lateral line. (**c**) 3D rendering of the eye with virtual transversal sectioning, 10 x magnification. (**d**) 3D rendering of the intestine, 3 x magnification. The spherical objects within the gut are artemia eggs.

Supplementary Figure 2 interactive 3D medaka anatomical atlas

ATLAS OF MEDAKA FISH

Oryzias latipes

Whole fish
Eyes
Nervous system
Cardiovascular system
Respiratory system
Digestive system
Urogenital system
Immune system
Skeleton
Muscles
Skin

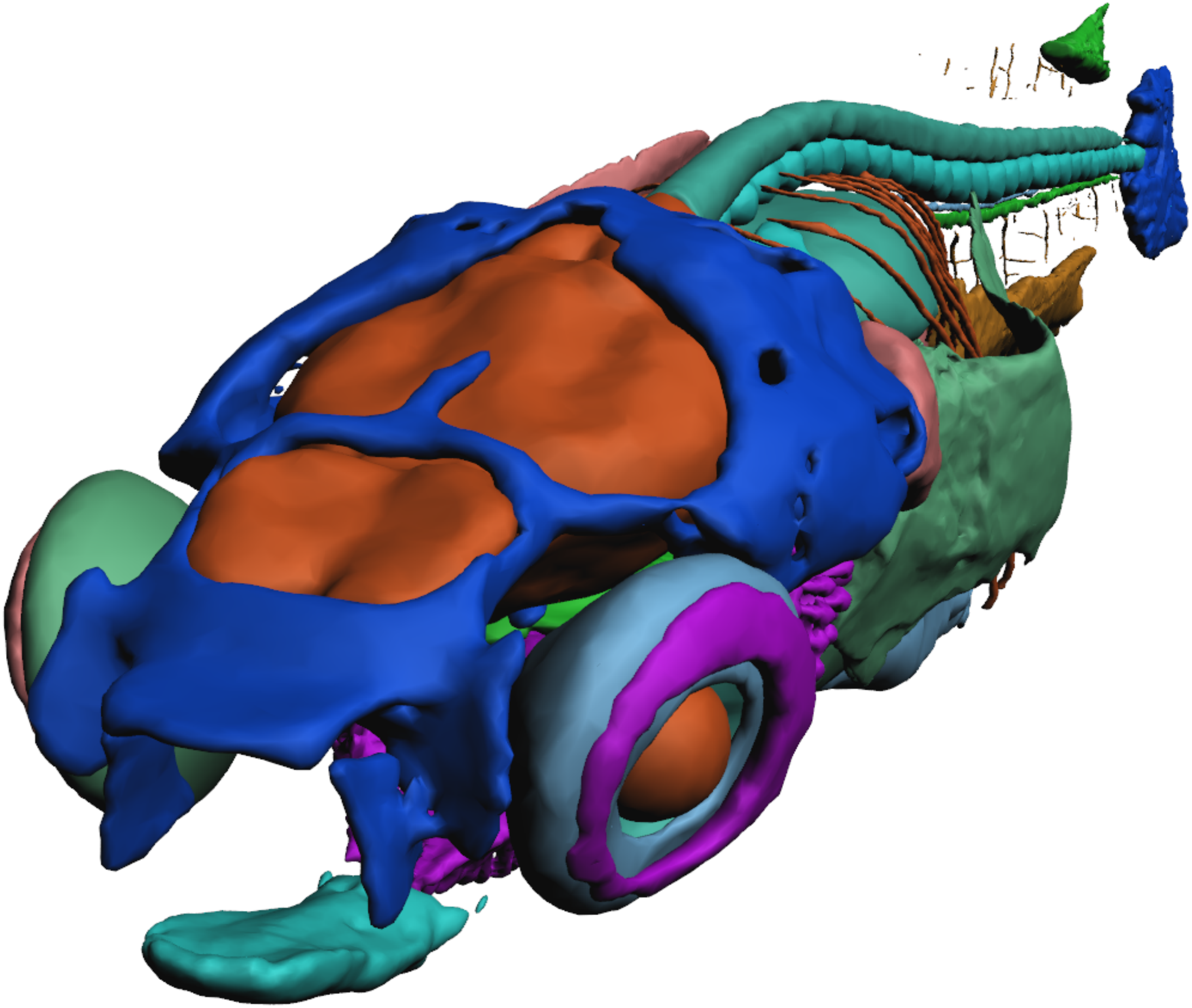

Quantitative morphometric analysis of adult teleosts by X-ray computed tomography

Venera Weinhardt, Roman Shkarin, Tobias Wernet, Joachim Wittbrodt, Tilo Baumbach and Felix Loosli

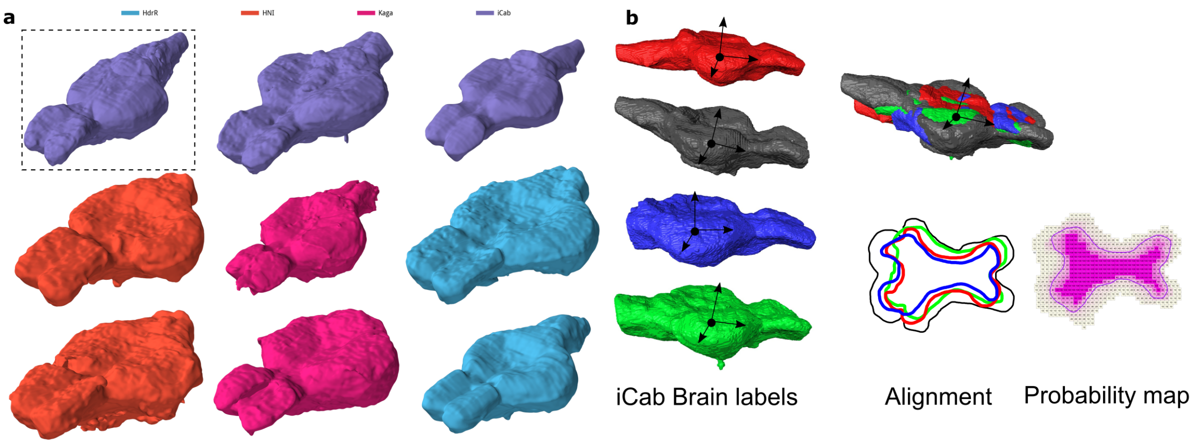

Supplementary Figure 3 Automated segmentation and organ probability maps

(a) 3D visualization of manually segmented iCab brain (indicated by hatched box) and eight automatically segmented brains (selected randomly from 4 inbred strains). All specimens are rescaled with respect to the reference model and therefore show only shape variation. **(b)** Schematic view of the pipeline for phenotypic analysis of the brain. Labels for each inbred strain (5 each) were aligned with respect to the common centre of mass (black dot) and coordinate system. The labels were averaged creating thus a 3D probability map for each inbred strain. For simplicity the figure shows the 2D case only. For the comparative analysis a cutoff of 60% probability value was used (magenta line on the probability map).

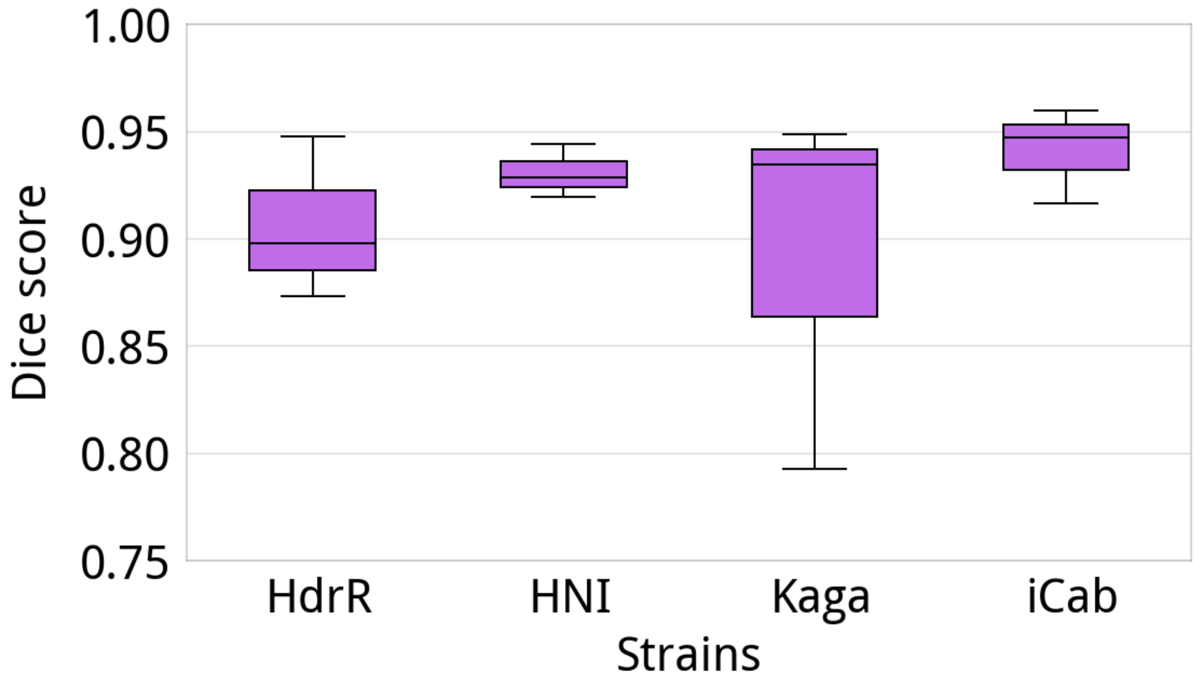

Supplementary Figure 4 Comparison of manual and automated segmentation

The similarity of manual and automated segmentation of brain for each strain measured by the dice similarity coefficient. Most of the automatically segmented labels have 90% and higher similarity to manual segmentation.

Supplementary Table 1 Tested fixatives and staining agents

	Concentration
Fixative	
paraformaldehyde	4%
glutaraldehyde	1%
Paraformaldehyde+ glutaraldehyde	4%+1%
Contrast agents	
Phosphotungstic acid	0.1, 0.3, 0.5, 1, 1.5, 3 and 5%
(Lugol's) iodine potassium iodine	0.1, 0.3, 0.5, 1, 1.5, 3 and 5%
Europium Chloride	0.1, 0.3, 0.5, 1, 1.5, 3 and 5%

Movies:

movie 1 Sagittal virtual sections of an adult medaka stained with PTA

movie 2 Manually segmented labels overlaid on the sagittal virtual sections of an adult medaka. The colours are the same as in the anatomical atlas. Labels for skin and muscles were excluded for better clarity.

movie 3 3D anatomical atlas of an adult medaka fish

movie 4 Use of interfaces in the 3D interactive atlas
